# International mobility of students in the medical disciplines from a comparative perspective

**DOI:** 10.3205/zma001327

**Published:** 2020-04-15

**Authors:** Martin Gartmeier, Maike Reimer, Johanna Huber, Nurith Epstein, Martin R. Fischer, Pascal O. Berberat

**Affiliations:** 1 Technical University of Munich, Faculty of Medicine, Hospital rechts der Isar, TUM Medical Education Center, Munich, Germany; 2 Bayrisches Staatsinstitut für Hochschulforschung und Hochschulplanung, Munich, Germany; 3 LMU Munich, Hospital University Munich, Institut für Didaktik und Ausbildungsforschung in der Medizin, Munich, Germany

**Keywords:** internship abroad, study abroad, internationalization, stay abroad, international mobility

## Abstract

**Objective: **We analyze the extent to which students of human, veterinary and dental medicine complete study-related stays abroad (frequency, type and duration of stays abroad and countries visited). Furthermore, we investigate the possible correlations between completed stays abroad and the duration of studies, the completion of a doctorate and entering professional life.

**Methods: **The data come from a written cross-sectional survey of 742 graduates of their respective study programs at Bavarian universities. The evaluation was carried out using descriptive and inferential statistical methods.

**Results: **Slightly more than half of the surveyed students completed study-associated stays abroad, with notable differences between the three study programs. The students most frequently completed internships abroad lasting an average of nine weeks. Switzerland was the most common country of destination for the stays abroad. Furthermore, there were no or only weak correlations between stays abroad, the duration of studies and progress towards a doctorate or the commencement of professional employment abroad. There were no correlations with the stress experienced as part of initial employment after graduation.

**Conclusion: **The results clearly indicate that stays abroad are quite usual for students in the medical disciplines and are almost standard in the study of human medicine. The selection of the countries visited indicates that the primary goal of the students' stays abroad is to deepen their competence with a view to later employment in their home country.

## Zusammenfassung

**Zielsetzung: **Wir analysieren das Ausmaß, in dem Studierende der Human-, Tier- und Zahnmedizin studienbezogene Auslandsaufenthalte absolvieren (Häufigkeit, Art und Dauer von Auslandsaufenthalten und besuchte Länder). Außerdem gehen wir der Frage nach, welche Zusammenhänge sich zwischen absolvierten Auslandsaufenthalte und der Studiendauer, der Absolvierung einer Promotion und dem Berufseinstieg zeigen.

**Methodik: **Die Daten stammen aus einer schriftlichen, querschnittlichen Befragung von 742 Absolvent/innen der entsprechenden Studiengänge an bayrischen Universitäten. Die Auswertung erfolgte durch deskriptive und inferenzstatistische Methoden.

**Ergebnisse: **Etwas mehr als die Hälfte der befragten Studierenden absolvierte studienassoziierte Auslandsaufenthalte, wobei sich starke Unterschiede zwischen den drei Studiengängen zeigten. Die Studierenden absolvierten am häufigsten Auslandspraktika, die im Schnitt neun Wochen dauerten. Am häufigsten war die Schweiz Zielland der Auslandsaufenthalte. Weiterhin zeigten sich keine bis schwache Zusammenhänge zwischen Auslandsaufenthalten, der Studiendauer und dem Fortschritt im Promotionsprozess oder der Aufnahme einer Berufstätigkeit im Ausland. Keine Zusammenhänge zeigten sich mit dem Belastungserleben im Rahmen der ersten Tätigkeit nach dem Studium.

**Schlussfolgerung: **Die Ergebnisse machen deutlich, dass Auslandsaufenthalte für Studierende medizinischer Fächer üblich, im Studium der Humanmedizin fast Standard sind. Die Auswahl der besuchten Länder deutet darauf hin, dass die Studierenden durch Auslandsaufenthalte primär das Ziel verfolgen, ihre Kompetenz im Hinblick auf eine spätere Berufstätigkeit im Inland zu vertiefen.

## 1. Introduction

Students of human, veterinary and dental medicine have many opportunities in the course of their studies to gain experience abroad – e.g., through internships or study stays abroad [1], [2]. This is not unique to the medical disciplines; the international mobility of students has become a feature of higher education policy across all disciplines [3]. However, various trends and developments justify particular consideration of the medical disciplines: Due to increasing individual mobility – in the form of tourism, migration, etc. – doctors also increasingly require intercultural skills in their home country [4]. For this reason, there is a call for greater consideration of global health (i.e., the consideration of health challenges in connection with globalization phenomena) and thus for increased attention to intercultural competence in medical studies [5], [6]. Universities are responding to this challenge by offering specialized global health courses [4], [7], by offering instruction in intercultural competence [8], and by promoting international student mobility [9]. 

Study-related stays abroad take various forms, e.g., as a study semester abroad or as internships abroad (in medicine, for example, as a clinical traineeship or as part of a practical year) [2], [6]. Various studies from the context of higher education research have addressed the extent, reasons for, and effects of international mobility of students [10], [11], [12] and graduates [13]. Other contributions are more narrative- [1] or program-oriented [6], [9], but to date there has been no comparative analysis focusing exclusively on the medical disciplines. These disciplines place special demands on students, e.g., with regard to the completion of compulsory courses during semester breaks. Insufficiency of time during studies is mentioned as an important factor with regard to internships abroad [14]. A systematic comparison of medical disciplines is therefore warranted. 

This paper focuses on the following questions:

1. To what extent and in what form do students in the medical disciplines complete study-related stays abroad?
2. What effects do these have on studies (focused on length of study and doctoral studies) and entry into professional employment (focused on working abroad and experience of stress)?

To answer these questions, we use data from a survey of graduates of the three medical study programs, which was conducted at the five medical faculties in Bavaria. In the following, we first present the current state of research with regard to the questions focused on here.

### 1.1. International mobility during medical studies

In 2011, the EU set a target for 20% of all university graduates in Europe to gain study-related experience abroad. The Joint Science Conference of the Federal Government and the Federal States (GWK^1^) has set even more ambitious mobility targets for Germany [15]: Every second German university graduate is expected to have gained discipline-related experience abroad during their studies. Research shows that the frequency as well as the type of stays abroad (e.g., internships, language courses or study phases) strongly depends on the particular discipline studied [16], [17]. This is self-evident since different courses of study offer different framework conditions with regard to the implementation of stays abroad [18] – e.g., due to different time constraints for students. In addition, existing comparative data only provide information on international mobility in the health sciences. Under this label, the medical disciplines (dental medicine, veterinary medicine and human medicine) are combined with related disciplines such as health sciences, health management or health care [19]. Other findings and studies only refer to students of human medicine [2], [20]. We therefore analyze the international mobility of medical students from a comparative perspective. To date, there are no conclusive empirical data in this regard. Thus, only general considerations may serve as a starting point for the generation of hypotheses: All medical degree programs feature a high degree of „regimentation“ – i.e., the proportion of compulsory courses is very high and the timetables of the various semesters are relatively fixed. Moreover, if credits earned abroad are not recognized, the loss of time associated with them is an obstacle to the completion of stays abroad. Furthermore, the study of dental medicine in particular features a high proportion of practical courses. These are often completed during the semester break and therefore compete with study-related stays abroad. Since this is not the case in human and veterinary medicine, we assume that students of dentistry spend fewer and shorter periods abroad. At the same time, it is above all doctors of human medicine who have to choose one of many possible specialist training courses after their studies. This suggests that they use lecture-free periods more frequently to familiarize themselves with various medical disciplines during (foreign) internships. Students of veterinary medicine probably fall midway between the other two medical disciplines. On this basis, the first group (A) of questions in our study can be formulated as follows:

- A1: How often do students in medical disciplines spend study-related periods abroad?
- A2: What types of stays abroad are completed?
- A3: How long do the completed internships abroad last?
- A4: What countries are visited in the context of internships abroad?

#### 1.2. Effects of stays abroad

Various authors argue that stays abroad promote students’ discipline-specific and interdisciplinary skills [21], but also have a positive influence on their personal development, career prospects [18], and employability [10]. Many studies examine the gains of stays abroad only in the form of subjective, sometimes very general assessments by students (such as “had an inspiring and exciting time?, “got to know another culture”, cf. [18], p. 80), or simply report on the experiences of students abroad in narrative form [22]. For students in the medical disciplines, too, the positive effects of a stay abroad are emphasized, e.g., by getting to know the way medicine is practised in different national contexts [1]. 

We concentrate here on objective study- and career-related effects. First of all, we are interested in whether stays abroad are associated with an extension of the duration of studies or a delay in completing the process of achieving a doctorate. Doctoral studies in medicine are often completed during the course of studies, which could lead to time conflicts in connection with stays abroad. Stays abroad could therefore slow down progress in gaining a doctorate. With regard to career development, we examine the question of whether graduates who are mobile are more likely to work abroad. A certain tendency among German physicians to migrate abroad was reported back in 2006 [23] although later studies do not confirm this [24]. We also examine whether internationally mobile graduates experience their first employment as less stressful. Studies show that students feel more self-confident after a stay abroad, that they claim to be more aware of their own strengths and weaknesses, and that they value their professional knowledge and cognitive skills more highly [18], [25]. The following specific questions are focused on here:

- B1: What effects do stays abroad have on studies (number of semesters studied and progress towards a doctorate)?
- B2: What effects do stays abroad have on entry into professional employment (initial employment in another country and experience of stress)?

## 2. Methods

### 2.1. The study

The current study is based on a survey of graduates in human medicine (HM), veterinary medicine (VM), and dental medicine (DM) from the five Bavarian faculties of medicine at the Friedrich-Alexander-University Erlangen-Nuremberg (HM and DM), the Ludwig-Maximilians-University Munich (HM, VM and DM), the Technical University Munich (HM only), the University Regensburg (HM and DM), and the Julius-Maximilians-University Würzburg (HM and DM). The survey was conducted in the winter of 2015/16 and was part of the Bavarian Graduate Panel [26]. It was targeted at a total of 1,900 individuals who had obtained a medical degree (third state examination) between April 1, 2014 and March 30, 2015. The survey was administered both online and as a paper-based questionnaire.

#### 2.2. Survey sample

The sample consisted of 742 participants, of which 479 (65%) had completed a degree in human medicine (HM), 155 (21%) in dental medicine (DM), and 108 (15%) in veterinary medicine (VM). 67% of the respondents were female and 28% male, while 5% gave no information regarding their gender. The gender distribution across the study programs was as follows: HM (♀ 62%, ♂ 32%, no answer 6%), VM (♀ 90%, ♂ 8%, no answer 2%) and DM (♀ 68%, ♂ 28%, no answer 4%). At the time of the survey, the respondents were on average 28.4 years old (*SD*=3.3), and the differences between the disciplines were small (HM: *M*=28.7, *SD*=3.22 / VM: *M*=27.7; *SD*=3.7 / DM: *M*=27.7; *SD*=3.2). 

#### 2.3. Operationalization and data analysis

In the survey, the respondents initially reported how many study-related stays abroad they had completed. In addition, the respondents were asked to provide more detailed information on their two longest stays abroad with regard to their type (study, internship, project work, language course, summer school, excursion/study trip, other study-related stay), their duration in weeks (free text item), and the country visited (free text item). In addition, various other data were analysed: field of study (HM, DM, VM), the total number of discipline-related semesters studied, the place of first professional employment (Germany or abroad) as well as the nationality of the interviewees (German/non-German) to account for the fact that for non-German citizens the move to another country is a priori more likely to occur even without a stay abroad during their studies. Furthermore, the experience of stress during the respondents‘ first job was investigated. Insofar as they felt these were applicable to their first professional employment, the respondents were asked to assess nine stress criteria: professional overload, work overload, heavy responsibility, time pressure, long working hours/overtime, too many on-call duties, too little time for patients, hierarchical structure, uncooperative working atmosphere. The items showed good internal consistency (Cronbach’s Alpha = .83) and were combined into one scale. Finally, respondents were asked about the stage of their doctoral studies. The question put was: “Have you undertaken doctoral studies?” The answer options were

1. “No, I do not intend to”;
2. “No, but I intend to do a doctorate”,
3. “Yes, but the doctorate is not yet completed” and
4. “Yes, the doctorate is already completed”.

Higher scores represent greater ambition/progress in the area of doctoral studies. All data analyses were performed using SPSS Version 24 software. In order to answer the research questions A1-A4, statistical parameters (*N, M, Min, Max, SD, Median*) were determined in the course of descriptive analyses. To answer questions B1 and B2 (effects of stays abroad), bivariate Pearson correlations were calculated. Furthermore, logistic regression analysis [27] was used to predict the dichotomous outcome variable ‘internship abroad’ (completed or not) based on various categorical and non-categorical predictors (e.g., weeks spent abroad). 

## 3. Results

### 3.1. Frequency of stays abroad (Question A1)

The focus is firstly on the question of how often students in medical disciplines complete study-related stays abroad. We present the stays separately according to the three medical disciplines and summarize stays during and immediately before/after the studies.

Table 1 (Tab. 1) shows the percentage of students who completed either none or at least one stay abroad (columns two and three). It also shows how many stays abroad were completed (columns four to eight). Students of dental medicine have the lowest and students of human medicine the highest level of international mobility. More than two-thirds of all students of human medicine completed a stay abroad. In the field of dental medicine, barely a quarter completed a stay abroad, and in veterinary medicine roughly half. Approximately a further third of students of human medicine completed three or even more stays abroad. This group includes less than one percent of students of dental medicine and slightly less than ten percent of students of veterinary medicine. 

#### 3.2. Types of stays abroad (Question A2)

Table 2 (Tab. 2) provides an overview of all stays abroad reported in the survey, sorted by discipline. The most frequent form of stays abroad were internships (almost 60%), followed by study visits (18%). The remaining 20% are distributed over various formats, including project work, language courses, summer schools, etc. It is clear that stays abroad were mainly used by students of human and veterinary medicine to gain practical experience (compared to studying at a foreign university). This pattern does not apply to students of dental medicine, who most often assigned their stays abroad to the category “other?. 

#### 3.3. Duration of internships abroad (Question A3)

In the following, internships completed abroad are examined in more detail. First of all, the focus is on the question of the length of the internships (Question A3). The following table provides an overview of the duration of all reported internships abroad, sorted by discipline and in total (see table 3 (Tab. 3)). 

The 742 respondents reported a total of 434 internships abroad. A comparison of the various disciplines shows that students of dential mediicine completed slightly shorter stays (approx. 5 weeks on average), whereas both human and veterinary medicine students spent an average of more than 9 weeks abroad for internship purposes. 

#### 3.4. Countries visited in the context of internships abroad (Question A4)

Table 4 (Tab. 4) provides an overview of the countries in which internships abroad were completed. Column three summarizes the values for all medical disciplines, while columns four to six report the discipline-specific frequencies. The countries are sorted in descending order in column three according to frequency of visit. Switzerland was by far the most popular country among the students surveyed. The most popular European destinations continue to be France, Spain, Italy, Austria and Ireland – in contrast to, for example, the Eastern and Southeast European countries. The list is dominated by a few European countries; only the USA, South Africa, Canada and Australia are similarly popular. South Africa, Tanzania, Ecuador, India, China, Nepal and Sri Lanka are the most popular developing countries^2^ among students. The comparison of the three medical disciplines shows no major differences in student preferences, and the numbers in columns five and six are relatively small. A particularity in veterinary medicine seems to be the choice of Australia as a destination country. In the field of dental medicine, no similar effects are visible. 

With regard to empirically verifiable effects of stays abroad, we continued to consider effects directly related to studies (duration of studies and progress towards a doctorate) as well as effects related to entering professional employment (working abroad and experiencing stress). 

#### 3.5. Study-related effects of stays abroad (Question B1)

Across all the medical disciplines, there was no correlation between the total number of completed stays abroad and the number of semesters studied (r=-0.06). With regard to the different disciplines, the following values were found: human medicine: r=- 0.03; dental medicine: r=- 0.22 (p<0.01); veterinary medicine: r=-0.08. Thus, only in dental medicine a weak negative but statistically significant correlation emerged.

Furthermore, we considered the correlative relationship between the completion of stays abroad and the admission or progress towards a doctorate. A weak but statistically significant correlation of r=0.20 (p<0.01) was found. The positive direction of this correlation is interesting – i.e., more frequent stays abroad are associated with greater progress toward a doctorate. This result was valid only for human medicine (r=0.20), not dental medicine (r=0.09) or veterinary medicine (r=-0.10). 

#### 3.6. Effects of stays abroad on starting employment (Question B2)

In addition, we examined whether students who have been internationally mobile as part of their studies more often pursued a professional activity abroad or were employed in international contexts (cf. 28). In the present sample, 555 (75%) individuals entered their first professional employment in Germany. On average, they had completed *M*=1.19 internships abroad (*SD*=1.31). In contrast, 156 (21%) individuals started their first job abroad, having completed an average of *M*=1.33 internships abroad (*SD*=1.42). 31 individuals (4%) had not yet taken up professional employment. A multivariate, logistic regression was used to address the question of how best to predict when a person would take up professional employment abroad. Three variables were examined as predictors: non-German nationality, the number of weeks spent abroad as part of studies, and the number of weeks spent abroad as part of internships. A test of the logistic regression model versus a model containing only the constant was statistically significant. Thus, based on the predictors, a reliable prediction could be made regarding the commencement of first employment in the home country or abroad (Chi square=30.19; *p*<0.01 with *df*=3). The value for Nagelkerke’s *R^2^* was 0.19 – indicating a relatively low degree of clarification of the target variables‘ variance. The percentage of cases successfully predicted by the model was about 80% overall (93% of those employed domestically and 36% of those employed abroad). The Wald criterion showed non-German nationality as a significant predictor for taking up employment abroad (*p*<0.01). In contrast, the number of weeks spent abroad for internships was not a significant predictor (*p*>0.05). The number of weeks spent abroad for study purposes was also not significantly linked to the taking up of employment abroad (*p*>0.05). The Exp(B) value showed an eightfold increase in the probability of students of non-German nationality to take up an employment abroad. 

Finally, we investigated the question of whether the stress experienced by the respondents during their first professional employment was related to the completion of stays abroad. The bivariate correlation regarding the experience of stress was r=-0.12. All other possible measures (weeks abroad for study or internship purposes) showed even lower correlations with the respondents’ experience of stress during their first professional employment. 

## 4. Discussion

This article has examined the international mobility of graduates of medical study programs. We here discuss the key results of this study in the context of existing research on study-related international mobility. For students of human medicine, stays abroad nowadays seem to be an integral part of their studies – in our sample, more than 70% completed at least one stay abroad (compared to 23% of dentists and 46% of veterinarians). Just under 30% of students of human medicine even complete three or more stays abroad. In quantitative terms, the mobility goals formulated by the EU and the GWK [15] in the area of medical disciplines are therefore being achieved. 

To put these figures into perspective, comparisons with other subjects are interesting: Students of economics and business administration show the highest level of international mobility in comparison with other academic disciplines [18]. In 2013, 34% of the students of economics were internationally mobile, and in 2015 the figure was 46%. In contrast, mobility in engineering tends to be at the lower end of the spectrum in a comparison of disciplines. Here 18% of students in 2013 and 24% in 2015 completed study-related stays abroad. Furthermore, the results reported here are of the same order of magnitude as the current figures from Störmann and Angstwurm [2]: Of the 554 physicians surveyed, 51% reported that they had completed at least part of their practical year abroad (the difference is plausible given the focus of stays abroad on the practical year). As previously mentioned, we could not find any sources that provided comparative information on international mobility in the various disciplines of medicine. Our results in this regard can therefore be considered exploratory and should be reviewed in the context of further studies. Our results support the assumption that the curricular and structural conditions in human medicine allow for stays abroad with relatively little loss of time and that such stays are therefore more frequently undertaken. In view of the high level of international mobility, it can also be assumed that a minimum level of international experience will become established as the “expected norm? among students [28].

The most common form of stays abroad in the medical disciplines were internships abroad, followed by study visits. This may indicate that in all medical disciplines, study phases or semesters abroad are less easily integrated into the temporal and structural framework of the courses than internships. In the field of dental medicine, 38% of the responses fell into the indeterminate category “other?. Based on the available data, we were unfortunately unable to clarify which specific stays abroad the students had completed that could not be described sufficiently well on basis of the given categories. 

According to our data, stays abroad lasted about nine weeks (HM & VM) and five weeks (DM) on average. Furthermore, stays abroad have little to do with further studies and entering professional employment: Beyond individual, relatively weak correlations, no demonstrable correlations were found with the duration of studies, progress toward a doctorate, or the stress experienced during first professional employment after graduation. Only non-German nationality proved to be a significant predictor of whether a student would take up first professional employment abroad. However, we were only able to consider the first one or two years after graduation. There may also be time-delayed effects where young doctors only seek a position abroad after several years of professional experience or completion of specialist training. Only in dental mediicine is there a low but significant negative correlation, which indicates that the duration of studies is extended by internships abroad. In tendency, it therefore seems to be more difficult in dental medicine than in other disciplines to reconcile stays abroad with regular studies.

With regard to the countries visited, our data showed that Switzerland, France and the USA were the most popular. With the exception of South Africa, the other top positions in the ranking were also occupied by first-world countries with very well-developed health systems and a western culture. In Switzerland and Austria, students scarcely encounter any language barriers. Given the effusive descriptions of the relevance of stays abroad in the education of “global citizens? [1] and the noble objectives of policy makers [15], the critical question is to what extent medical students actually gain new cultural insights and understanding of global health problems during their internships abroad. It seems that medical students primarily use stays abroad to extend their medical knowledge and skills with a view to seamless transition into professional life. Current data show that students in other disciplines prefer similar destinations to medical students for their stays abroad [29]. 

One limitation of the present study is that not all of the three medical degree programs are offered at all of the Bavarian universities where the students surveyed had completed their studies. Thus, the variables “university location? and “course of study? are possible confounded. It is possible, for example, that different universities create differently favorable conditions for studying abroad. In addition, a possible limitation of the significance of the study is that not all graduates of the relevant years were reached by the survey. It may be that precisely those graduates who had taken up their first employment abroad were not reached. This factor could be related to the variables investigated here. Furthermore, our data cannot provide any insight into the motives, evaluations, and experiences that medical students associate with completing a stay abroad. It would be of interest in further studies to investigate the influence of different motivational factors on the organization and evaluation of stays abroad.

The available results provide insight into the significance of stays abroad for students in the medical disciplines: It appears that such stays are increasingly becoming the norm for students, especially in the field of human medicine. At the same time, it cannot be determined on the basis of the available data which measurable positive/negative effects are associated with completing stays abroad. Further research in this direction is therefore recommended. 

## Notes

^1^ Die Gemeinsame Wissenschaftskonferenz des Bundes und der Länder (GWK)

^2^ The basis for designating these countries as “developing countries” is the DAC list for the reporting years 2018-2020, see https://www.bmz.de/de/ministerium/zahlen_fakten/oda/hintergrund/dac_laenderliste/index.html

## Competing interests

The authors declare that they have no competing interests. 

## Figures and Tables

**Table 1:**
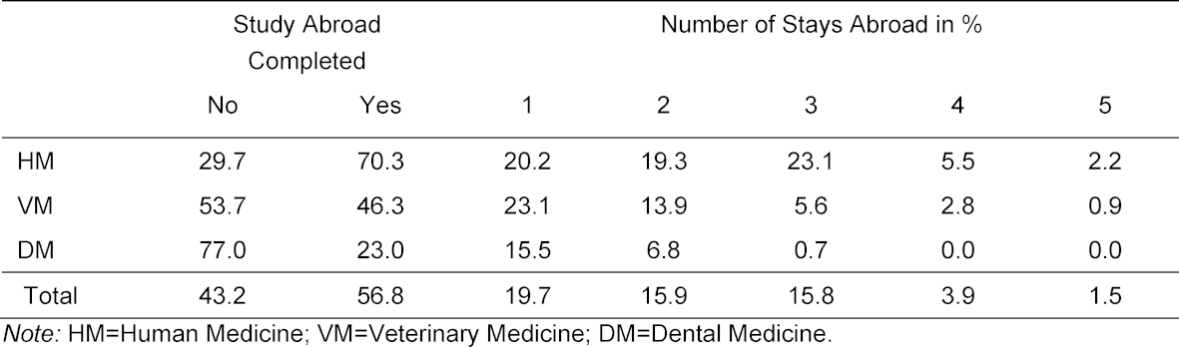
Frequency of discipline-related stays abroad before, during and after studies in the various medical disciplines

**Table 2:**
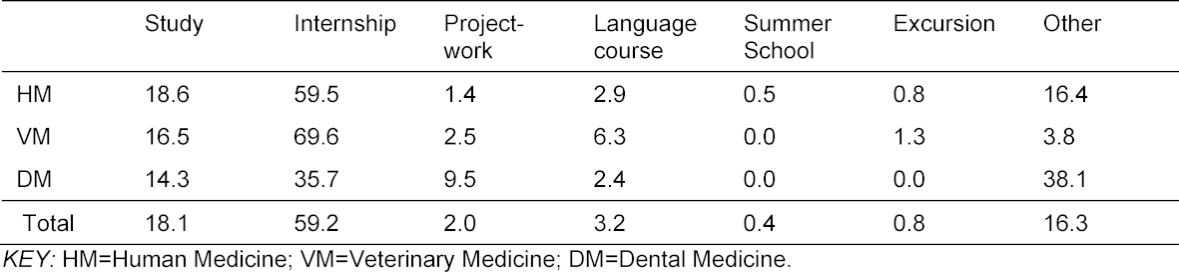
Types of discipline-related stays abroad in the various medical disciplines (in %)

**Table 3:**
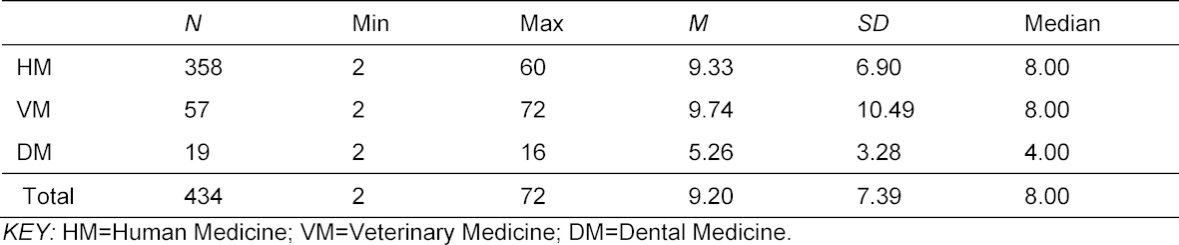
Duration of internships abroad in the various medical disciplines in weeks

**Table 4:**
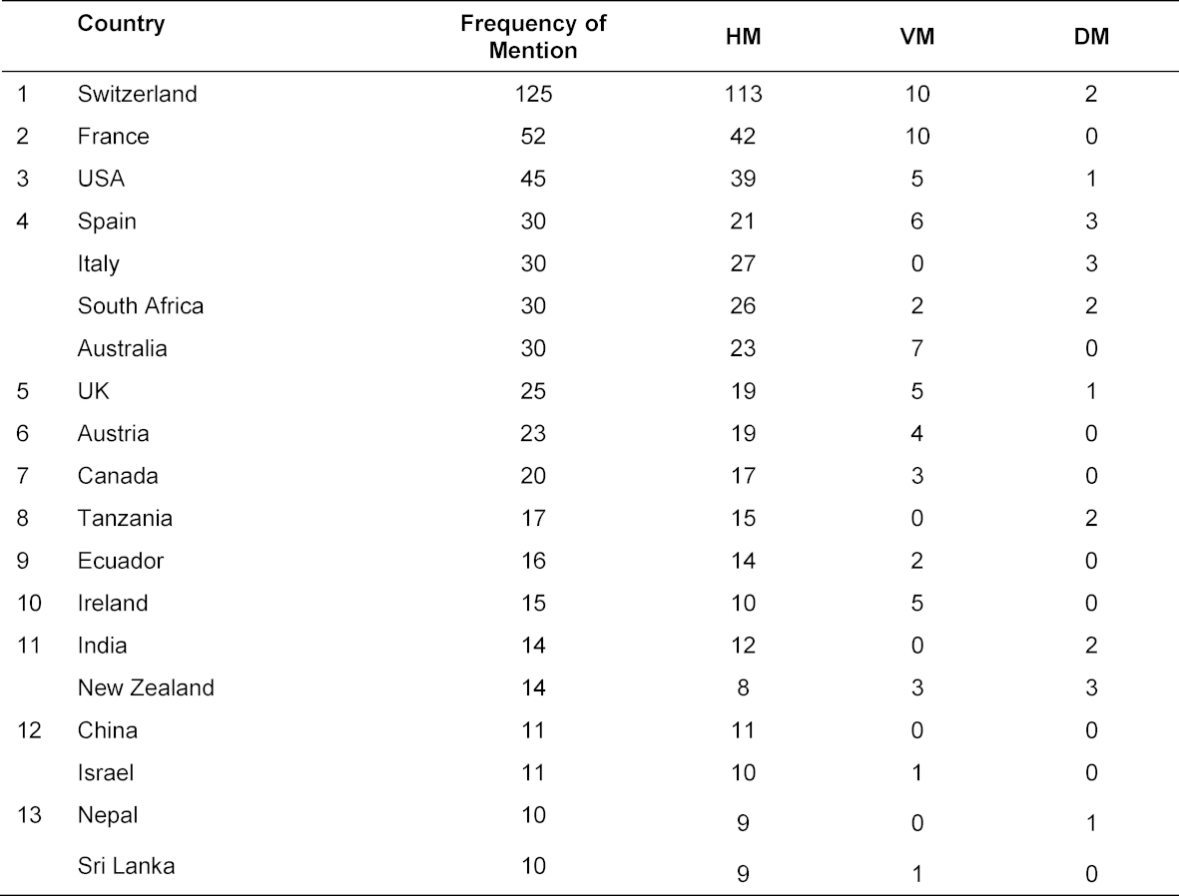
Countries visited as part of medical internships abroad (general and per medical specialty)
